# Daratumumab Improves Bone Turnover in Relapsed/Refractory Multiple Myeloma; Phase 2 Study “REBUILD”

**DOI:** 10.3390/cancers14112768

**Published:** 2022-06-02

**Authors:** Evangelos Terpos, Ioannis Ntanasis-Stathopoulos, Efstathios Kastritis, Evdoxia Hatjiharissi, Eirini Katodritou, Evangelos Eleutherakis-Papaiakovou, Evgenia Verrou, Maria Gavriatopoulou, Alexandros Leonidakis, Kyriaki Manousou, Sosana Delimpasi, Panagiotis Malandrakis, Marie-Christine Kyrtsonis, Maria Papaioannou, Argiris Symeonidis, Meletios-Athanasios Dimopoulos

**Affiliations:** 1 Department of Clinical Therapeutics, School of Medicine, National and Kapodistrian University of Athens, 11528 Athens, Greece; johnntanasis@med.uoa.gr (I.N.-S.); ekastritis@med.uoa.gr (E.K.); evelepapa@med.uoa.gr (E.E.-P.); mgavria@med.uoa.gr (M.G.); panosmalan@med.uoa.gr (P.M.); mdimop@med.uoa.gr (M.-A.D.); 2 First Department of Internal Medicine, School of Medicine, AHEPA University Hospital, Aristotle University of Thessaloniki, 54124 Thessaloniki, Greece; ehatjiharissi@gmail.com (E.H.); papaioam@auth.gr (M.P.); 3 Department of Hematology, Theagenio Cancer Hospital, 54639 Thessaloniki, Greece; aimatologiki@thegenio.gov.gr (E.K.); everrou@gmail.com (E.V.); 4 Health Data Specialists S.A., 11525 Athens, Greece; a.leonidakis@heads-research.com (A.L.); k.manousou@heads-research.com (K.M.); 5 Bone Marrow Transplantation Unit and Department of Hematology, Evangelismos Hospital, 10676 Athens, Greece; sodeli@yahoo.com; 6 First Department of Propedeutic Internal Medicine, School of Medicine, National and Kapodistrian University of Athens, 11527 Athens, Greece; kyrtsoni@med.uoa.gr; 7 Hematology Division, Department of Internal Medicine, School of Medicine, University of Patras, 26334 Patras, Greece; argisym@upatras.gr

**Keywords:** multiple myeloma, daratumumab, bone metabolism, biomarker, clinical trial

## Abstract

**Simple Summary:**

Multiple myeloma (MM) is characterized by the presence of deregulated bone metabolism. Restoring bone turnover is essential for patients with MM. We prospectively evaluated the impact of the anti-CD38 monoclonal antibody daratumumab on markers of bone remodeling among patients with relapsed/refractory MM. Overall, daratumumab improved bone turnover by favoring bone formation.

**Abstract:**

Biomarkers of bone turnover in serum are suggestive of bone dynamics during treatment in multiple myeloma (MM). We evaluated the role of daratumumab on bone remodeling among patients with relapsed/refractory MM in the prospective, open-label, phase 2 study REBUILD. Daratumumab was administered according to the approved indication. A total of 33 out of 57 enrolled patients completed 4 months of treatment. The median percent change from baseline to 4 months in C-terminal cross-linking telopeptide of type 1 collagen (CTX) (primary endpoint) was 3.9%, with 13 (39.4%) and 11 (33.3%) patients showing at least 20% and 30% reduction in CTX levels, respectively. The median percent decrease from baseline to 4 months in tartrate resistant acid phosphatase 5b (TRACP-5b) levels (co-primary endpoint) was 2.6%, with 10 (30.3%) and 6 (18.2%) patients showing at least 20% and 30% reduction in TRACP-5b levels, respectively. However, the changes in these markers of bone catabolism were not statistically significant. Furthermore, the levels of osteocalcin, bone-specific alkaline phosphatase and procollagen type-I N-pro-peptide (bone formation markers) increased from baseline to 4 months (secondary endpoints) by 18.4%, 92.6% and 10.2%, respectively. Furthermore, the median levels of dickkopf-1 and C-C motif ligand-3 showed a significant decrease at 4 months by 17.5% and 16.0%, respectively. In conclusion, daratumumab improved bone turnover by inducing bone formation and reducing osteoblast inhibition.

## 1. Introduction

A better understanding of the molecular pathogenesis of multiple myeloma (MM) has led to improved therapeutic approaches and patient outcomes. However, MM remains an incurable malignancy [1]. The development of osteolytic bone disease is a key clinical characteristic of MM, and it is attributed to the disruption of the delicate balance between bone formation and bone resorption [2]. Bisphosphonates, especially zoledronic acid, and denosumab, a monoclonal antibody binding to the receptor activator of nuclear factor kappa-Β ligand (RANKL), are the mainstay of treatment for MM-related bone disease [3]. Furthermore, anti-myeloma drugs, such as the proteasome inhibitors bortezomib and carfilzomib, have shown a positive effect on bone health [4,5,6,7,8]. Consequently, research into agents that combine anti-myeloma activity with positive effects on bone metabolism is of high importance [9,10].

During the last decade, monoclonal antibodies targeting CD38 (daratumumab, isatuximab) have improved patient outcomes significantly, and they have been incorporated into the therapeutic algorithm of MM both for newly diagnosed patients and in the relapsed/refractory setting [11]. Although the safety and efficacy profiles of these drugs are well established, their role in bone metabolism is rather vague. Preclinical studies have shown that CD38 has a key role in the induction of osteoclast formation and bone resorption [12]. Monocytes and early progenitors of osteoclasts express CD38 on their cellular membrane; therefore, daratumumab, which is an anti-CD38 agent, may inhibit osteoclastogenesis [13].

Taking into consideration all of the above, we scheduled a phase 2 clinical study to determine the effect of daratumumab, (Janssen Biotech, Inc. Horsham, PA, USA) on bone disease and bone metabolism in patients with advanced relapsed/refractory MM who received daratumumab monotherapy.

## 2. Materials and Methods

The aim of this study was to evaluate the effect of daratumumab monotherapy on bone disease and bone metabolism in patients with relapsed/refractory MM. The patients should have been previously exposed to at least one proteasome inhibitor and lenalidomide. Bone turnover was assessed with the determination of serum indicators of bone catabolism and anabolism during treatment with daratumumab [14].

### 2.1. Study Design

This was a prospective, multicenter, non-comparative, open-label, phase II study (ClinicalTrials.gov identifier: NCT03475628, accessed on 25 April 2022). Daratumumab was administrated in patients with relapsed and/or refractory MM with at least two prior lines of therapy, including a proteasome inhibitor and lenalidomide (Celgene Corporation, Summit, NJ, USA), according to the first approved drug label in Greece. Daratumumab was not approved for patients with newly diagnosed MM in Greece at the time of study design. Daratumumab was administered at 16 mg/kg intravenously weekly for the first two cycles of treatment, biweekly for the next 4 cycles of treatment and monthly thereafter. Patients received treatment until disease progression, physician decision, unacceptable toxicity, withdrawal of consent, or death (whichever occurred first). Survival status and data on subsequent anti-myeloma treatment post daratumumab were also collected. The study was approved by the institutional review board, and it was conducted in accordance with the Declaration of Helsinki and the International Conference on Harmonization for Good Clinical Practice.

### 2.2. Patient Selection

This study included adult patients with documented relapsed and/or refractory MM and measurable disease in serum (M-protein, free light chains) and/or urine (U-protein). Patients should have received at least two lines of prior anti-myeloma treatment including lenalidomide and a proteasome inhibitor, and they should have documented evidence of disease progression according to the International Myeloma Working Group Criteria [14]. Eligible individuals showed a Karnofsky Performance Status score of at least 70, alanine aminotransferase level ≤2.5 times the upper limit of normal (ULN), adequate renal function as defined by a creatinine clearance of at least 30 mL/min by Chronic Kidney Disease Epidemiology Collaboration (CKD-EPI), absolute neutrophil count of at least 1.0 × 10^9^/L, platelet count of at least 75 × 10^9^/L or 50 × 10^9^/L in patients with more than 50% plasma cell invasion in the bone marrow, and hemoglobin above 7.5 g/dL. All patients provided written informed consent before enrolment in the study.

### 2.3. Study Endpoints

The study’s primary goal was to compare baseline values to changes in indicators of bone catabolism C-telopeptide of collagen type 1 (CTX) and tartrate-resistant acid phosphatase 5b (TRACP-5b) after 4 months of daratumumab initiation.

The secondary endpoints of the study were: (i) changes in serum indices of bone anabolism (osteocalcin [OC], bone alkaline phosphatase [bALP] and procollagen type I N-terminal pro-peptide [PINP]) after 4, 8 and 12 months (or treatment completion) on daratumumab compared with baseline; (ii) reduction in serum indices of bone catabolism (CTX and TRACP-5b) after 8 and 12 months (or treatment completion) on daratumumab compared with baseline; (iii) reduction in circulating osteoclast regulators RANKL, RANKL/osteoprotegerin (OPG) ratio and CC-motif ligand-3 (CCL-3) after 4, 8 and 12 months (or treatment completion) on daratumumab compared with baseline; (iv) reduction in circulating osteoblast inhibitors dickkopf-1 (Dkk-1) and sclerostin (SOST) after 4, 8 and 12 months (or treatment completion) on daratumumab compared with baseline; (v) changes in bone mineral density (BMD) of the lumbar spine or the hip assessed by dual-energy X-ray absorptiometry (DXA) after 6 and 12 months of therapy; (vi) overall response rate (ORR) according to the International Myeloma Working Group criteria [15], progression free survival (PFS), time to next treatment (TtNT) and overall survival (OS); (vii) skeletal-related events (SRE) encompassing pathological fractures, need for bone radiotherapy or bone surgery, and spinal cord compression.

### 2.4. Evaluation of Bone Remodeling

Serum markers of bone metabolism were examined at baseline and then every 2 months of therapy until the completion of 12 months of treatment or treatment completion, whichever occurred first. Patient serum was separated within 4 h following vein puncture and stored at 0 °C until the day of measurement. An enzyme-linked immunosorbent assay (ELISA) was applied for the evaluation of serum indices according to manufacturer’s instructions: sRANKL (Biomedica Medizinprodukte, Gesellschaft GmbH & Co KG, Wien, Austria) with intra- and inter-assay coefficient of variability (CV) <5% and <9%, respectively; OPG (Biomedica Medizinprodukte) with intra- and inter-assay CV <10% and <8%, respectively; CCL-3 (Quantikine, R&D systems, Minneapolis, MN, USA) with intra- and inter-assay CVs of <3% and <7%, respectively; Dkk-1 (Biomedica Medizinprodukte) with intra- and inter-assay CV <8% and <12%, respectively; TRACP-5b (BoneTRAP, Immunodiagnostic Systems, Boldon, Tyne & Wear, UK) with intra- and inter-assay CV <13.9% and <9.2%, respectively; CTX (Serum Croslaps, Immunodiagnostic Systems) with intra- and inter-assay CV <3% and <10.9%, respectively; bALP (Metra BAP, Quidel Corporation, San Diego, CA, USA) with intra- and inter-assay CV <5.8%and <7.6%, respectively, P1NP (Abbexa Ltd., Cambridge, UK) with intra- and inter-assay CVs of <10%; and OC (N/MID Osteocalcin, Immunodiagnostic Systems Nordic A/S, Herlev, Denmark), with intra- and inter-assay CV <2.2% and <5.1%, respectively. Serum SOST levels were determined using a sandwich-type ELISA by Biomedica Laboratory (Wien, Austria); the detection limit was 0.2 ng/mL (8.9 pmol/L); the standard range was set from 0.33 to 5.4 ng/mL (15–240 pmol/L); and the CV for intra-assay was 4–6% and inter-assay was 5–7%. All samples from the same patient were measured on the same ELISA plate.

### 2.5. Statistical Analysis

The primary efficacy population included all eligible patients who received at least one dose of daratumumab and had available results for bone resorption markers at baseline and at 4 months post-treatment initiation. The full analysis set included all eligible patients who received at least one dose of daratumumab. Efficacy analyses were performed in the primary efficacy population (i.e., all eligible patients who received at least one daratumumab dose and had available results for bone resorption markers at baseline and at 4 months post-treatment onset). The statistical analysis was performed using SAS^®^ statistical analysis software (v. 9.4) (SAS Institute, Cary, NC, USA). Summary statistics are presented for categorical variables based on frequency tables. Regarding continuous variables, descriptive statistics (median, Q1, Q3, values) were used. The Kaplan-Meier method was applied for all time to event analyses. To further evaluate the changes in biomarkers over time until 12 months post-treatment onset, linear repeated measures models were fitted. In the final model, visit (i.e., cycle) was included as a fixed effect and the log-transformed absolute biomarker values at each timepoint were considered as dependent variables.

## 3. Results

### 3.1. Patient Characteristics

**Figure 1:**
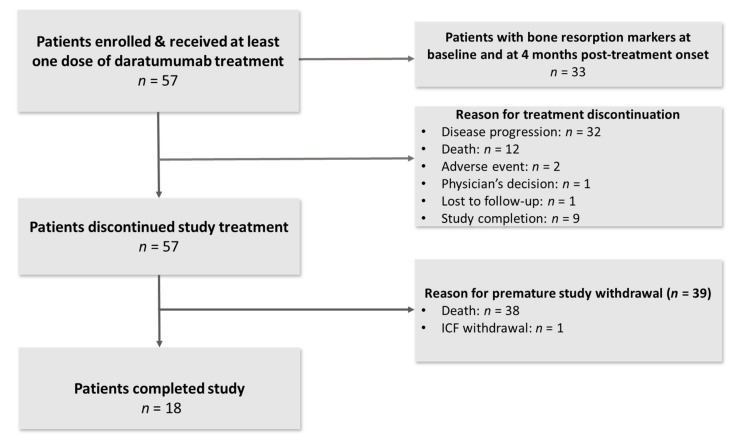
Study flowchart.

From 8 March 2018 to 26 February 2020, a total of 57 patients were enrolled across six sites in Greece (Figure 1). The median age of the enrolled patients was 73 years (range 52–87 years). Twenty-six patients were males (46%). The median number of prior lines of therapy was three (range 2–9), whereas 41 patients (72%) were refractory to their last line of therapy. All patients performed a baseline assessment for myeloma bone disease. Forty-five patients (79%) had at least one lytic bone lesion. Baseline patient characteristics are presented in detail in Table 1.

The enrolled patients received a median of six cycles (range 1–34) of treatment with daratumumab. The median follow up was 10.5 months (Q1–Q3: 3.8–19.5). Reasons for treatment discontinuation included disease progression (*n* = 32, 56.1%), death (*n* = 12, 21.1%), study completion (*n* = 9, 15.8%), adverse events (*n* = 2, 3.5%) and physician’s decision due to inadequate depth of response (*n* = 1, 1.8%). Thirteen (22.8%) patients received bisphosphonates along with daratumumab monotherapy.

### 3.2. Impact of Daratumumab on Bone Metabolism

Among the study participants, 33 had available results for bone resorption markers at baseline and at 4 months post-treatment onset. All 24 patients with no bone resorption markers at 4 months post-treatment onset discontinued treatment prior to 4 months.

The median percentage drop in CTX levels from baseline to 4 months was 3.9%, with 13 (39.4%) and 11 (33.3%) patients presenting at least 20% and 30% decrease in CTX levels, respectively. The median percentage decrease in TRACP-5b during the same time period was 2.6%, whereas 10 (30.3%) and 6 (18.2%) individuals showed at least 20% and 30% decrease in TRACP-5b levels, respectively.

Among patients with at least partial response (PR) at 4 months, the median percentage change from baseline in CTX and TRACP-5b levels was −1.3% and −2.6%, respectively. The median percentage changes for patients without a response at 4 months were 5.3% and −7.2% for CTX and TRACP-5b, respectively.

Overall, there were no statistically significant percent reductions in CTX and TRACP-5B at any measured timepoint up to 12 months compared with baseline. However, the highest proportion of patients with CTX reduction of 30% or greater was reported at 10 and 12 months post-treatment onset (50.0% and 57.1%, respectively). The greatest proportion of patients with TRACP-5b reduction of at least 30% was observed at 6 months following the start of treatment with daratumumab (37.5%). Furthermore, the greatest proportion of patients with a RANKL and RANKL/OPG reduction of at least 30% was observed at 4 months post-treatment onset (30.3% and 36.4%, respectively).

The levels of the bone formation markers bALP, OC, and PINP increased from baseline to 4 months; more specifically, the median percentage increase was 18.4%, 92.6% and 10.2%, respectively. The median rise in biomarker levels of bone anabolism from baseline to 4 months among patients with at least PR was 25.3% for bALP, 146.0% for OC, and 15.7% for PINP, respectively; the equivalent increases in patients without a response were 18.3%, 15.6%, and −7.3%. The changes in OC reached statistical significance, whereas the changes in bALP and PINP showed only a trend to significance in the PR subgroup. The changes in biomarkers of bone anabolism were not statistically significant in the non-responders subgroup.

Overall, a significant increase in median values of bALP was observed at 2 (22.2%, *p* < 0.001), 4 (18.4%, *p* = 0.020), 10 (29.3%, *p* = 0.049) and 12 months (22.5%, *p* = 0.049) compared with baseline. At 10 and 12 months after therapy, the proportion of patients with a bALP rise of at least 30% was highest (50.0% each). A significant increase in median values of OC was observed at all measured timepoints post-treatment initiation (97.6% [*p* < 0.001], 92.6% [*p* < 0.001], 109.1% [*p* = 0.004], 267.2% [*p* < 0.001], 274.5% [*p* = 0.007], and 297.1% [*p* = 0.003], at 2, 4, 6, 8, 10 and 12 months, respectively). At 8 months after starting daratumumab, the proportion of patients with OC rise of at least 30% became highest (77.8%). A significant increase in the median value of PINP was observed at 8 months compared with baseline (39.9%, *p* = 0.012). The greatest proportion of patients with PINP increase of at least 30% was observed at 8 (55.6%) and 12 (50.0%) months on study.

There were no significant changes in serum SOST levels at any of the examined timepoints compared with baseline. However, the greatest proportion of patients with a SOST reduction of at least 30% was observed at 10 (35.7%) and 12 (42.9%) months post-study entry.

A significant decrease in median values of DKK1 was observed at 2 (10.2%, *p* < 0.001), 4 (17.5%, *p* < 0.001), 6 (21.7%, *p* = 0.004), 8 (27.6%, *p* = 0.021), 10 (36.6%, *p* = 0.002) and 12 (38.3%, *p* < 0.001) months post daratumumab initiation. The greatest proportion of patients with DKK1 reductions of at least 30% was observed at 10 (64.3%) and 12 (78.6%) months compared with baseline.

A significant decrease in median values of CCL3 was observed at 12 months compared with baseline (26.1%, *p* = 0.017). The greatest proportion of patients with CCL3 reduction of at least 30% was observed at 10 (50%) and 12 (50%) months after beginning treatment with daratumumab.

Table 2 shows the results of the repeated measures models using all study assessments up to 12 months for all biomarkers of bone resorption and bone formation examined. Significant increases in OC and decreases in DKK1 and CCL3 were consistently observed over time. Similar results were shown among 33 patients included in the primary efficacy analysis (Table S1).

Regarding the assessment of bone mineral density, no significant changes were observed at 12 months compared with baseline in T- (*p* = 0.781) and Z-score (*p* = 0.074). No skeletal-related events (SREs) were reported during the study period.

### 3.3. Efficacy and Survival

Among the 33 patients included in the primary efficacy population, the overall response rate (ORR) (at least PR) was 63.6% (*n* = 21). One patient (3.0%) achieved complete remission (CR), seven patients (21.2%) achieved a very good partial remission (VGPR) and 13 individuals (39.4%) showed a PR. The median (95% confidence interval-CI) PFS was 9.3 (6.7–15.3) months, whereas the median (95% CI) OS was 21.2 (12.7-not reached) months.

**Figure 2:**
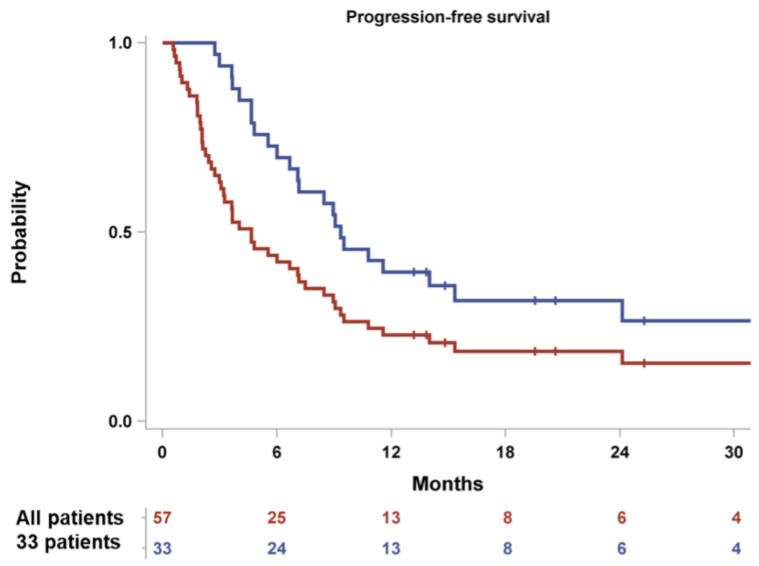
Kaplan–Meier curves of PFS for the enrolled patients (*n* = 57) and the primary analysis cohort (*n* = 33).

**Figure 3:**
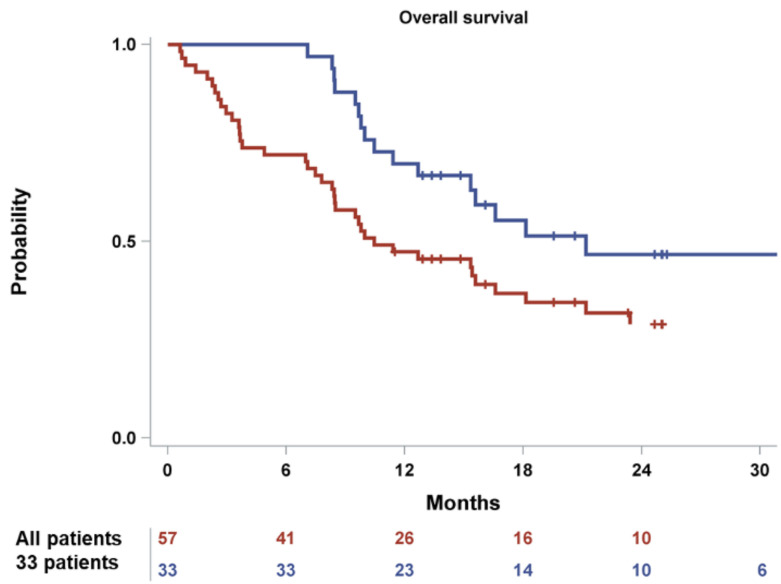
Kaplan–Meier curves of OS for the enrolled patients (*n* = 57) and the primary analysis cohort (*n* = 33).

Among the whole study population (*n* = 57) the median PFS and OS were 4.7 (3.0–7.2) and 10.5 (8.3–16.6) months, respectively. The Kaplan-Meier estimates for 6- and 12-month PFS rates were 42.1% (95% CI: 29.2, 54.4) and 22.8% (95% CI: 13.0, 34.3) (Figure 2). The Kaplan-Meier estimates for 12-month and 24-month OS rates were 47.36% and 28.9% (95% CI: 16.8, 42.1), respectively (Figure 3). Furthermore, 28 patients (49.1%) initiated a subsequent treatment during the study period. The Kaplan–Meier estimate for median TtNT was 7.10 (95% CI: 3.80, 9.10) months.

### 3.4. Safety

The most common grade 3 or 4 non serious adverse events were anemia (*n* = 15, 26.3%), neutropenia (*n* = 8, 14%) and thrombocytopenia (*n* = 8, 14%). Overall, 19 patients (33.3%) reported a serious adverse event. The most prevalent major adverse effects were lower respiratory tract infections which occurred in four patients (7%). In total, 17.5% of the patients had an adverse drug reaction to daratumumab. The serious adverse events related to daratumumab were febrile neutropenia grade 3 (*n* = 1), lower respiratory tract infections grade 3–5 (*n* = 4) and bronchospasm grade 4 (*n* = 1).

## 4. Discussion

Herein, we provide novel data from the first clinical study in the literature to assess bone health in individuals with relapsed/refractory MM receiving daratumumab. Daratumumab is an anti-CD38, human IgG1κ monoclonal antibody, which is abundantly expressed not only on malignant plasma cells but also on natural killer (NK) cells and B- and T-lymphocyte subsets [16,17]. CD38 has a multifaceted function and acts both as an activator of intracellular signaling cascades and as an ectoenzyme regulating intracellular nicotinamide adenine dinucleotide (NAD+) levels [17,18]. Daratumumab leads myeloma cells to death via both Fc-dependent immune effector pathways and direct effects [17,19,20]. Furthermore, daratumumab has an immunomodulatory effect by counteracting CD38-positive immunosuppressive lymphoid and myeloid cells [16,17,21]. In our study, daratumumab showed a favorable effect on serum indices of bone metabolism.

Daratumumab did not result in statistically significant differences in serum levels of indicators of bone catabolism CTX and TRACP-5b. This finding is in line with the non-significant changes in the RANKL/OPG ratio over time, as indicated by the repeated measures model analysis. In general, patients with MM have an elevated RANKL/OPG ratio. High values of RANKL/OPG have been associated with an increased burden of myeloma bone disease as well as poor patient survival [22]. A favorable effect of daratumumab on bone resorption was more pronounced among patients who received treatment for at least 6 months. Preclinical studies have used flow cytometry to show that CD38 is expressed on monocytes and early progenitors of osteoclasts but not on mature osteoclasts [13]. Therefore, daratumumab may inhibit bone resorption by targeting immature osteoclasts and preventing osteoclastogenesis [13]. In this context, daratumumab may also restore the osteoclast-induced immunosuppressive T-cell phenotype in the myeloma bone marrow milieu by reducing the levels of galectin-9 and a proliferation-induced ligand (APRIL) secreted by osteoclasts [23].

In addition to the above, daratumumab showed a favorable effect on bone formation by increasing the serum levels of bALP, OC and PINP. The anabolic benefit was greater among responders and those with a prolonged duration of treatment. This is in line with the anabolic effect of carfilzomib on myeloma bone health in patients with relapsed/refractory MM [5,24]. Although CD38 is not highly expressed by osteoblasts [13], our results point towards an indirect effect of daratumumab inducing bone formation, which is mediated by both an anti-myeloma effect and a decrease in inhibitors of osteoblast activity. The regulation of osteoclasts and osteoblasts in the bone marrow microenvironment is in a fine-tuned balance [25,26]. The immunomodulatory effect of daratumumab along with the reduction in myeloma load may allow for bone microenvironment equilibrium and the production of new bone [25].

Regarding the effect of daratumumab on osteoblast inhibitors, a significant decrease in DKK1 levels was evident, which became more pronounced over time. DKK1 is a soluble, extracellular antagonist of the Wnt signaling pathway, which plays a key role in the regulation of bone mass [27,28]. DKK1 levels are elevated in patients with MM, which in turn suppresses the differentiation of osteoblasts in favor of osteoclasts and promotes myeloma-related bone destruction [28,29]. DKK1 upregulation leads to increased SOST expression, whereas both molecules have a synergistic inhibitory impact on bone growth [30]. SOST is expressed by both myeloma cells and osteocytes, and it acts as a negative regulator of the Wnt pathway and, consequently, bone formation [31,32,33]. Patients with MM and increased SOST levels present with impaired bone turnover, advanced disease stage and adverse prognosis [34,35]. However, SOST levels did not show any significant changes with daratumumab treatment in our study. In the relapsed/refractory setting, carfilzomib has resulted in a reduction in both DKK1 and SOST serum levels [5].

Furthermore, daratumumab resulted in a significant reduction in CCL3 levels, which was consistently demonstrated over time. Similar findings have been reported with carfilzomib [5]. CCL3 or macrophage inflammatory protein protein-1 alpha (MIP-1 alpha) is a pro-inflammatory chemokine with multiple effects [36]. Increased serum levels of CCL3 in patients with MM have been correlated with severe bone damage owing to the induction of osteoclast maturation, as well as poor patient prognosis [37,38]. Furthermore, CCL3 may downregulate the osteogenic transcription factor osterix, which in turn results in decreased OC expression and osteoblast malfunction [39].

Our study did not reveal any effect of daratumumab on bone mineral density, whereas no SREs were documented. Although changes in bone mineral density may be a surrogate for fracture outcomes [40], the follow-up time in our study (6 and 12 months) may be considered short for sizeable changes in bone mineral density indices to occur. Data from both clinical trials and real-world studies indicate that most SREs are reported during the first year following MM diagnosis [41,42]. An improvement in biomarkers markers of bone turnover has been associated with a reduced risk of SREs [43].

Daratumumab had a median PFS of 4.7 months and a median OS of 10.5 months in patients with relapsed/refractory MM with a median of 3 (range 2–9) previous lines of treatment. Although cross-trial comparisons should be made with caution, we showed a slightly prolonged median PFS and an inferior median OS compared with other clinical trials of daratumumab monotherapy (SIRIUS, GEN501) [44,45,46,47]. These differences may be attributed to distinct baseline patient characteristics (frailty, myeloma stage) enrolled in each study. The toxicity profile of the study drug was manageable and in line with the previously published studies (hematologic toxicity, infusion-related reactions, infections) [44,45,46,47], whereas no new major adverse events were reported.

The main drawback of our study is the small number of patients who participated. The evaluation of secondary study endpoints and subgroup analyses may have been underpowered due to the limited number of patients in each category. Furthermore, the timing and duration of exposure to bone-directed therapies (bisphosphonates, denosumab) prior to daratumumab initiation may have a residual effect on the kinetics of serum bone markers and bone health. Last but not least, an evaluation of bone marrow biopsies would be important for the histological assessment of the daratumumab effect on bone remodeling.

## 5. Conclusions

In conclusion, daratumumab showed a positive effect on bone metabolism in patients with relapsed/refractory MM primarily by reducing osteoblast inhibition and inducing bone formation. Prolonged exposure to daratumumab improved bone health indices. Restoring bone health in patients with MM by supporting the bone microenvironment to return to equilibrium is critical for improving quality of life. Further studies are deemed necessary in order to confirm the favorable effect of daratumumab in conjunction with antiresorptive and targeted drugs on bone strength and patient outcomes.

## Figures and Tables

**Table 1:** Baseline characteristics of 57 enrolled patients.

Variable	Patient Disposition
Male sex, *n* (%)	26 (45.6%)
Karnofsky Performance Status, median (range)	90 (70–100)
Caucasian race, *n* (%)	57 (100%)
Age at enrolment, median (range), years	73 (52–87)
Age at diagnosis, median (range), years	68 (44–83)
Time from diagnosis to enrolment, median (Q1–Q3), years	4.6 (2.9–7.7)
Number of prior lines of therapy, median (range)	3 (2–9)
Prior ASCT, *n* (%)	7 (12.3%)
Refractory to the last line of therapy, *n* (%)	41 (71.9%)
Refractory to PI, *n* (%)	37 (64.9%)
Refractory to IMiD, *n* (%)	47 (82.5%)
Refractory to both PI and IMiD, *n* (%)	36 (63.2%)
No Lytic Bone Lesions, *n* (%)	12 (21.1%)
1–3 Lytic Bone Lesions, *n* (%)	6 (10.5%)
4–10 Lytic Bone Lesions, *n* (%)	9 (15.8%)
More than 10 Lytic Bone Lesions, *n* (%)	30 (52.6%)
ISS stage I, *n* (%)	13 (22.8%)
ISS stage II, *n* (%)	24 (42.1%)
ISS stage III, *n* (%)	20 (35.1%)
IgG Myeloma, *n* (%)	29 (50.9%)
IgA Myeloma, *n* (%)	9 (15.8%)
Kappa Light Chain Myeloma, *n* (%)	8 (14.0%)
Lamda Light Chain Myeloma, *n* (%)	8 (14.0%)
IgD Myeloma, *n* (%)	1 (1.8%)
Biclonal Myeloma, *n* (%)	1 (1.8%)
CTX (ng/mL), median (Q1, Q3)	1.0 (0.4, 2.0)
TRACP-5B (U/L), median (Q1, Q3)	1.9 (1.1, 2.8)
RANKL (pmol/L), median (Q1, Q3)	0.1 (0.0, 0.2)
bALP (μg/L), median (Q1, Q3)	8.5 (6.6, 11.2)
OC (ng/mL), median (Q1, Q3)	2.7 (1.2, 6.3)
PINP (pg/mL), median (Q1, Q3)	521.0 (307.1, 1071.2)
SOST (pmol/L), median (Q1, Q3)	34.2 (21.0, 64.3)
Dkk1 (pmol/L), median (Q1, Q3)	58.1 (36.0, 100.6)
CCL3 (ng/mL), median (Q1, Q3)	35.4 (19.2, 57.5)

ASCT, Autologous Stem-Cell Transplant; IMiD, Immunomodulatory Drug; ISS, international staging system; n, number of patients; PI, Proteasome Inhibitor; Q1, first quartile; Q3, third quartile; CTX: C-telopeptide of collagen type 1; TRACP-5b: tartrate-resistant acid phosphatase 5b; OC: osteocalcin; bALP: bone alkaline phosphatase; PINP: procollagen type I N-terminal pro-peptide; RANKL: receptor activator of nuclear factor kappa-Β ligand; CCL3: CC-motif ligand-3; Dkk1: dickkopf-1; SOST: sclerostin.

**Table 2:** Overview of the changes in biomarker values over time using repeated measures models.

	Baseline	2 Months	4 Months	6 Months	8 Months	10 Months	12 Months
**bALP (μg/L)**							
*n*	56	43	33	24	18	14	14
Median absolute change from baseline (Q1, Q3)		2.1 (−0.7, 5.1)	1.4 (−0.8, 3.6)	1.7 (−1.8, 4.3)	1.8 (−1.5, 5.2)	2.0 (−0.5, 6.3)	2.0 (−0.2, 4.8)
*p*-value for absolute change ^a^		**0.005**	**0.045**	0.129	0.129	0.055	0.215
**Osteocalcin (ng/mL)**							
*n*	56	43	33	24	18	14	14
Median absolute change from baseline (Q1, Q3)		2.0 (−1.2, 6.7)	1.5 (−1.3, 6.7)	1.1 (−3.2, 6.3)	4.2 (1.2, 8.7)	3.1 (−0.1, 7.5)	4.4 (0.3, 9.7)
*p*-value for absolute change ^a^		**0.023**	0.061	**0.039**	**0.001**	**0.014**	**0.008**
**PINP (pg/mL)**							
*n*	56	43	33	24	18	14	14
Median absolute change from baseline (Q1, Q3)		35.2 (−167.3, 198.1)	34.4 (−155.5, 225.8)	82.7 (−98.8, 266.0)	375.7 (36.0, 1785.0)	149.5 (−103.8, 1880.4)	376.5 (−80.7, 1539.3)
*p*-value for absolute change ^a^		0.686	0.348	0.810	**0.010**	0.074	0.085
**CTX (ng/mL)**							
*n*	56	43	33	24	18	14	14
Median absolute change from baseline (Q1, Q3)		−0.0 (−0.2, 0.3)	0.0 (−0.2, 0.3)	0.1 (−0.0, 0.5)	0.0 (−0.3, 0.7)	−0.1 (−0.6, 0.2)	−0.1 (−1.1, 0.0)
*p*-value for absolute change ^a^		0.728	0.695	0.990	0.522	0.179	0.060
**TRACP-5B (U/L)**							
*n*	56	43	33	24	18	14	14
Median absolute change from baseline (Q1, Q3)		−0.2 (−0.7, 0.7)	−0.1 (−0.5, 0.7)	−0.1 (−0.9, 0.5)	0.2 (−0.5, 1.0)	0.3 (−0.6, 0.9)	0.1 (−0.4, 0.6)
*p*-value for absolute change ^a^		0.753	0.273	0.720	0.277	0.780	0.969
**RANKL (pmol/L)**							
*n*	56	43	33	24	18	14	14
Median absolute change from baseline (Q1, Q3)		0.0 (−0.0, 0.1)	0.0 (−0.0, 0.1)	0.0 (−0.0, 0.1)	0.0 (−0.0, 0.2)	0.0 (0.0, 0.1)	0.1 (0.0, 0.2)
*p*-value for absolute change ^a^		0.089	0.149	0.268	0.111	0.201	**0.028**
**RANKL/OPG ratio**							
*n*	56	43	33	24	18	14	14
Median absolute change from baseline (Q1, Q3)		0.0 (−0.0, 0.0)	0.0 (−0.0, 0.0)	0.0 (−0.0, 0.0)	0.0 (−0.0, 0.0)	0.0 (−0.0, 0.0)	0.0 (−0.0, 0.0)
*p*-value for absolute change ^a^		0.112	0.269	0.275	0.327	0.204	0.085
**SOST (pmol/L)**							
*n*	56	43	33	24	18	14	14
Median absolute change from baseline (Q1, Q3)		−1.2 (−12.8, 6.0)	2.8 (−13.9, 16.5)	−6.3 (−26.5, 8.0)	−4.1 (−42.1, 1.5)	0.5 (−40.4, 19.5)	−4.0 (−33.4, 52.0)
*p*-value for absolute change ^a^		0.971	0.363	0.878	0.836	0.877	0.589
**DKK1 (pmol/L)**							
*n*	56	43	33	24	18	14	14
Median absolute change from baseline (Q1, Q3)		−7.9 (−21.2, −1.5)	−8.7 (−29.2, −2.1)	−11.9 (−24.4, −2.4)	−13.7 (−25.7, −6.5)	−16.2 (−36.8, −9.7)	−17.0 (−33.6, −11.1)
*p*-value for absolute change ^a^		0.079	0.191	**0.042**	**0.049**	**0.006**	**0.002**
**CCL3 (ng/mL)**							
*n*	56	43	33	24	18	14	14
Median absolute change from baseline (Q1, Q3)		−2.4 (−6.0, 9.3)	−3.6 (−13.7, 1.7)	−5.7 (−28.2, 0.8)	−7.8 (−32.7, 5.3)	−10.4 (−25.9, 3.0)	−8.3 (−24.8, −0.7)
*p*-value for absolute change ^a^		0.366	0.068	**0.021**	**0.007**	**0.008**	**0.006**

bALP, bone-specific alkaline phosphatase; CCL3, CC-motif ligand-3; CTX, C-telopeptide of collagen type 1; DKK1, Dickkopf-1; *n*, number of patients; OPG, Osteoprotegerin; PINP, Procollagen type I N Propeptide; Q1, first quartile; Q3, third quartile; RANKL, Receptor Activator of Nuclear factor Kappa-B Ligand; SOST, Sclerostin; TRACB-5b, Tartrate-Resistant Acid Phosphatase-5b; ^a^ estimated using a linear repeated measures model with biomarker log-transformed values at each timepoint as the depended variable and visit (i.e., cycle) as fixed effect.

## Data Availability

Manuscript data are available upon reasonable request from the corresponding author.

## Supplementary Materials

The following are available online at https://www.mdpi.com/article/10.3390/cancers14112768/s1, Table S1. Overview of the changes in biomarker values over time using repeated measures models; primary analysis population (*n* = 33).
