# Supplementary material for: Daratumumab Improves Bone Turnover in Relapsed/Refractory Multiple Myeloma; Phase 2 Study “REBUILD”
Source: Cancers (Basel). 2022 Jun 2;14(11):2768. doi: 10.3390/cancers14112768 (PMC9179322; doi:10.3390/cancers14112768)
Supplement: Supplementary file 1 [file cancers-14-02768-s001.zip › cancers-1697975-supplementary.pdf]

cancers MDPI

Supplementary Materials

Daratumumab Improves Bone Turnover in Relapsed/Refractory Multiple Myeloma; Phase 2 Study "REBUILD"

Evangelos Terpos [1,*], Ioannis Ntanasis-Stathopoulos [1], Efstathios Kastritis [1], Evdoxia Hatjiharissi [2], Eirini Katodritou [3], Evangelos Eleutherakis-Papaiakovou [1], Evgenia Verrou [3], Maria Gavriatopoulou [1], Alexandros Leonidakis [4], Kyriaki Manousou [4], Sosana Delimpasi [5], Panagiotis Malandrakis [1], Marie-Christine Kyrtsonis [6], Maria Papaioannou [2], Argiris Symeonidis [7] and Meletios-Athanasios Dimopoulos [1]

1 Department of Clinical Therapeutics, School of Medicine, National and Kapodistrian University of Athens, 11528 Athens, Greece; johnntanasis@med.uoa.gr (I.N.-S.); ekastritis@med.uoa.gr (E.K.); evelepapa@med.uoa.gr (E.E.-P.); mgavria@med.uoa.gr (M.G.); panosmalan@med.uoa.gr (P.M.); mdimop@med.uoa.gr (M.-A.D.)
2 First Department of Internal Medicine, School of Medicine, AHEPA University Hospital, Aristotle University of Thessaloniki, 54124 Thessaloniki, Greece; ehatjiharissi@gmail.com (E.H.); papaioam@auth.gr (M.P.)
3 Department of Hematology, Theagenio Cancer Hospital, 54639 Thessaloniki, Greece; aimatologiki@thegenio.gov.gr (E.K.); everrou@gmail.com (E.V.)
4 Health Data Specialists S.A., 11525 Athens, Greece; a.leonidakis@heads-research.com (A.L.); k.manousou@heads-research.com (K.M.)
5 Bone Marrow Transplantation Unit and Department of Hematology, Evangelismos Hospital, 10676 Athens, Greece; sodeli@yahoo.com
6 First Department of Propedeutic Internal Medicine, School of Medicine, National and Kapodistrian University of Athens, 11527 Athens, Greece; kyrtsoni@med.uoa.gr
7 Hematology Division, Department of Internal Medicine, School of Medicine, University of Patras, 26334 Patras, Greece; argisym@upatras.gr
* Correspondence: eterpos@med.uoa.gr; Tel.: +30-(213)-216-2846

Table S1. Overview of the changes in biomarker values over time using repeated measures models; primary analysis population (*n* = 33).

	Baseline	2 Months	4 Months	6 Months	8 Months	10 Months	12 Months
**bALP (μg/L)**							
n	33	33	33	24	18	14	14
Median biomarker value (Q1, Q3)	8.3 (6.6,10.1)	10.2 (7.9,15.2)	9.5 (7.9,11.9)	9.7 (8.1,11.8)	8.9 (7.6,13.7)	10.4 (8.0,13.7)	10.2 (8.1,12.3)
Median percent change from baseline (Q1, Q3)		25.0 (-0.6,54.6)	18.4 (-8.3,45.5)	22.0 (-14.8,62.1)	27.3 (-16.8,71.5)	29.3 (-4.9,99.1)	22.5 (-2.9,78.6)
Median absolute change from baseline (Q1, Q3)		2.1 (-0.1,5.5)	1.4 (-0.8,3.6)	1.7 (-1.8,4.3)	1.8 (-1.5,5.2)	2.0 (-0.5,6.3)	2.0 (-0.2,4.8)
p-value for absolute change[a]		**0.018**	**0.140**	0.280	0.255	0.114	0.363
**Osteocalcin (ng/ml)**							
n	33	33	33	24	18	14	14
Median biomarker value (Q1, Q3)	2.6 (1.2,7.0)	5.3 (1.8,11.3)	5.7 (2.1,13.5)	5.6 (2.3,15.1)	9.1 (5.1,14.0)	6.6 (3.8,13.0)	8.3 (3.8,10.4)
Median percent change from baseline (Q1, Q3)		108.2 (-24.9,256.3)	92.6 (-18.1,352.1)	109.1 (-44.3,361.9)	267.2 (98.1,571.8)	274.5 (-0.9,804.2)	297.1 (29.2,447.2)
Median absolute change from baseline (Q1, Q3)		1.6 (-1.2,5.6)	1.5 (-1.3,6.7)	1.1 (-3.2,6.3)	4.2 (1.2,8.7)	3.1 (-0.1,7.5)	4.4 (0.3,9.7)

	Baseline	2 Months	4 Months	6 Months	8 Months	10 Months	12 Months
p-value for absolute change[a]		0.111	0.118	0.075	**0.004**	**0.026**	**0.016**
**PINP (pg/ml)**							
n	33	33	33	24	18	14	14
Median biomarker value (Q1, Q3)	661.1 (302.6,974.3)	626.0 (424.2,1163.4)	747.2 (404.4,1174.9)	604.2 (292.7,1608.1)	1345.9 (636.5,3937.7)	1294.0 (551.3,3202.5)	1416.4 (655.3,2552.0)
Median percent change from baseline (Q1, Q3)		11.6 (-18.2,65.0)	10.2 (-16.9,54.5)	10.2 (-24.6,82.0)	39.9 (6.9,264.9)	20.3 (-16.6,168.4)	34.0 (-19.5,162.1)
Median absolute change from baseline (Q1, Q3)		56.7 (-95.5,316.8)	34.4 (-155.5,225.8)	82.7 (-98.8,266.0)	375.7 (36.0,1785.0)	149.5 (-103.8,1880.4)	376.5 (-80.7,1539.3)
p-value for absolute change[a]		0.616	0.484	0.920	**0.025**	0.120	0.135
**CTX (ng/ml)**							
n	33	33	33	24	18	14	14
Median biomarker value (Q1, Q3)	1.3 (0.4,2.1)	0.8 (0.4,2.3)	0.7 (0.4,2.3)	0.9 (0.3,2.4)	0.5 (0.3,2.4)	0.5 (0.3,0.9)	0.4 (0.3,0.5)
Median percent change from baseline (Q1, Q3)		-9.1 (-33.9,19.5)	3.9 (-38.6,30.1)	20.4 (-11.2,51.5)	6.6 (-52.5,41.6)	-23.7 (-66.8,59.8)	-33.9 (-79.4,0.9)
Median absolute change from baseline (Q1, Q3)		-0.0 (-0.2,0.3)	0.0 (-0.2,0.3)	0.1 (-0.0,0.5)	0.0 (-0.3,0.7)	-0.1 (-0.6,0.2)	-0.1 (-1.1,0.0)
p-value for absolute change[a]		0.501	0.461	0.705	0.365	0.128	**0.045**
**TRACP-5B (U/L)**							
n	33	33	33	24	18	14	14
Median biomarker value (Q1, Q3)	1.9 (1.3,3.3)	1.9 (1.3,2.7)	2.0 (1.5,2.9)	1.6 (1.3,2.7)	2.1 (1.5,2.9)	1.8 (1.5,2.9)	1.8 (1.5,2.3)
Median percent change from baseline (Q1, Q3)		-7.4 (-32.0,46.3)	-2.6 (-23.6,31.7)	-2.9 (-41.5,34.4)	10.3 (-18.6,45.0)	20.3 (-31.3,78.9)	5.7 (-21.6,38.6)
Median absolute change from baseline (Q1, Q3)		−0.2 (−0.6,0.7)	−0.1 (−0.5,0.7)	−0.1 (−0.9,0.5)	0.2 (−0.5,1.0)	0.3 (−0.6,0.9)	0.1 (−0.4,0.6)
p-value for absolute change[a]		0.760	0.806	0.319	0.696	0.761	0.547
**RANKL (pmol/L)**							
n	33	33	33	24	18	14	14
Median biomarker value (Q1, Q3)	0.1 (0.0,0.2)	0.1 (0.1,0.2)	0.1 (0.1,0.2)	0.1 (0.1,0.2)	0.1 (0.1,0.2)	0.1 (0.1,0.2)	0.2 (0.1,0.2)
Median percent change from baseline (Q1, Q3)		−3.4 (−34.5,149.2)	20.7 (−32.9,100.9)	19.3 (−13.8,102.4)	74.0 (−26.9,194.6)	32.1 (5.9,253.8)	83.0 (23.4,121.2)
Median absolute change from baseline (Q1, Q3)		−0.0 (−0.0,0.1)	0.0 (−0.0,0.1)	0.0 (−0.0,0.1)	0.0 (−0.0,0.2)	0.0 (0.0,0.1)	0.1 (0.0,0.2)
p-value for absolute change[a]		0.318	0.468	0.647	0.283	0.422	0.068
**RANKL/OPG ratio**							
n	33	33	33	24	18	14	14
Median biomarker value (Q1, Q3)	0.02 (0.00,0.03)	0.01 (0.00,0.03)	0.01 (0.00,0.03)	0.01 (0.00,0.03)	0.01 (0.00,0.02)	0.01 (0.00,0.03)	0.01 (0.00,0.02)
Median percent change from baseline (Q1, Q3)		23.9 (−34.1,137.9)	3.8 (−39.3,147.1)	43.6 (−19.8,129.8)	48.3 (−38.6,270.3)	32.2 (−29.7,240.5)	57.1 (−15.9,179.5)
Median absolute change from baseline (Q1, Q3)		0.0 (−0.0,0.0)	0.0 (−0.0,0.0)	0.0 (−0.0,0.0)	0.0 (−0.0,0.0)	0.0 (−0.0,0.0)	0.0 (−0.0,0.0)
p-value for absolute change[a]		0.246	0.670	0.620	0.652	0.402	0.179
**SOST (pmol/L)**							
n	33	33	33	24	18	14	14

	**Baseline**	**2 Months**	**4 Months**	**6 Months**	**8 Months**	**10 Months**	**12 Months**
Median biomarker value (Q1, Q3)	33.8 (23.8,65.2)	26.9 (23.2,60.7)	44.2 (27.5,57.4)	31.9 (26.7,50.2)	39.3 (27.4,51.4)	42.0 (26.2,68.1)	53.4 (25.5,85.1)
Median percent change from baseline (Q1, Q3)		−5.8 (−25.8,18.2)	2.7 (−32.2,69.3)	−18.3 (−32.9,37.3)	−14.6 (−43.1,6.5)	1.5 (−40.5,39.9)	−16.5 (−48.8,187.7)
Median absolute change from baseline (Q1, Q3)		−4.5 (−12.8,4.4)	2.8 (−13.9,16.5)	−6.3 (−26.5,8.0)	−4.1 (−42.1,1.5)	0.5 (−40.4,19.5)	−4.0 (−33.4,52.0)
p-value for absolute change[a]		0.717	0.554	0.721	0.696	0.973	0.738
**Dkk1 (pmol/L)**							
n	33	33	33	24	18	14	14
Median biomarker value (Q1, Q3)	58.4 (38.3,104.3)	51.7 (32.8,68.1)	49.3 (36.9,71.2)	45.8 (30.2,63.0)	46.2 (32.2,60.2)	37.4 (31.9,54.4)	35.6 (27.7,49.7)
Median percent change from baseline (Q1, Q3)		−14.1 (−47.4,-5.8)	−17.5 (−42.5,−4.9)	−21.7 (−45.1,−6.3)	−27.6 (−35.6,−11.7)	−36.6 (−52.8,−16.9)	−38.3 (−54.1,−30.4)
Median absolute change from baseline (Q1, Q3)		−9.5 (−22.7,−2.7)	−8.7 (−29.2,−2.1)	−11.9 (−24.4,−2.4)	−13.7 (−25.7,−6.5)	−16.2 (−36.8,-9.7)	−17.0 (−33.6,−11.1)
p-value for absolute change[a]		**0.019**	0.061	**0.012**	**0.015**	**0.001**	**<0.001**
**CCL3 (ng/ml)**							
n	33	33	33	24	18	14	14
Median biomarker value (Q1, Q3)	34.2 (16.3,46.8)	27.9 (20.8,37.2)	26.6 (18.2,37.2)	21.8 (17.9,33.0)	21.9 (16.2,26.4)	20.5 (12.7,27.2)	21.3 (12.0,34.8)
Median percent change from baseline (Q1, Q3)		−6.4 (−16.5,55.9)	−16.0 (−34.4,6.0)	−22.5 (−48.5,2.1)	−22.0 (−61.9,34.7)	−34.1 (−66.1,7.7)	−26.1 (−63.3,−1.9)
Median absolute change from baseline (Q1, Q3)		−2.0 (−6.0,7.5)	−3.6 (−13.7,1.7)	−5.7 (−28.2,0.8)	−7.8 (−32.7,5.3)	−10.4 (−25.9,3.0)	−8.3 (−24.8,−0.7)
p-value for absolute change[a]		0.572	0.229	0.078	**0.025**	**0.024**	**0.020**

n, number of patients; NA, Not applicable, Q1, first quartile; Q3, third quartile, a estimated using a linear repeated measures model with biomarker log-transformed values at each timepoint as the depended variable and visit (i.e., cycle) as fixed effect.
